# Active and Passive Immunization of Syrian Hamsters with An Attenuated SARS-CoV-2 Protects against New Variants of Concern

**DOI:** 10.21203/rs.3.rs-2227555/v1

**Published:** 2022-11-11

**Authors:** Tony Wang, Charles Stauft, Prabhuanand Selvaraj, Felice D'agnillo, Clement Meseda, Kotou Sangare, Cyntia Pedro, Shufeng Liu, Christopher Lien, Jerry Weir, Matthew Starost

## Abstract

Detection of secretory antibodies in the airway is highly desirable when evaluating mucosal protection by a vaccine against a respiratory virus like the severe acute respiratory syndrome coronavirus 2 (SARS-CoV-2). We show that a single intranasal delivery of an attenuated SARS-CoV-2 (Nsp1-K164A/H165A) induced both mucosal and systemic IgA and IgG in Syrian hamsters. Interestingly, either active or passive immunization of hamsters with Nsp1-K164A/H165A offered protection against heterologous challenge with variants of concern (VOCs) including Delta, Omicron BA.1, and Omicron BA.2.12.1. Among challenged animals, Nsp1-K164A/H165A vaccination specifically reduced viral loads in the respiratory tract and suppressed infection-induced macrophage accumulation and MX1 upregulation in the lung. The absence of variant-specific mucosal and systemic antibodies was associated with breakthrough infections, particularly of the nasal cavity following challenges with Omicron isolates. Together, our study demonstrates that an attenuated nasal vaccine may be developed to boost mucosal immunity against future SARS-CoV-2 VOCs.

## Introduction

The early success of the two mRNA vaccines against SARS-CoV-2 boasted up to 95% efficacy against the most severe disease outcomes[1,2]. Nevertheless, the emergence of new virus variants has resulted in reduced vaccine effectiveness against symptomatic COVID-19. Since the emergence of the Omicron variant of concern (VOC) in late 2021, SARS-CoV-2 Omicron sub-lineages (e.g., BA.1, BA.2, BA.3, BA.4, BA.5, etc.) have gradually replaced previously circulating variants worldwide[3]. Owing to increased immune evasion, infection with Omicron has led to large numbers of hospitalizations and deaths[4–6]. Vaccine effectiveness against Omicron after two BNT162b2 doses was modest at 2 to 4 weeks and fell to nearly zero after 6 months or more[7–9]. A third and fourth mRNA vaccine booster did improve efficacy against Omicron to 60–70%[10–12], but the durability of protection was less than impressive, with a mean 30-day rate of decay in neutralizing antibody titers of nearly 20% against BA.4/5[13].

Several studies have suggested that COVID-19 vaccine performance might be improved if mucosal immunity can be enhanced. For example, a recent study demonstrated that combining systemic mRNA vaccination with mucosal adenovirus-S immunization induced strong neutralizing antibody (nAb) responses against both the ancestral virus and the Omicron BA.1.1 variant in mice. By contrast, systemic mRNA vaccination alone induced weak respiratory mucosal neutralizing antibody responses[14]. Earlier in another proof-of-concept study, an intranasal vaccination with nonadjuvanted spike subunit protein following intramuscular mRNA vaccinations in mice elicited protective mucosal immunity via memory T/B cells and IgA that significantly lowered viral load in the upper and lower airways and prevented disease and death from a lethal SARS-CoV-2 challenge[15]. These findings highlight the importance of understanding the mucosal immunogenicity and efficacy of next-generation nasal vaccines [16].

Of the many vaccines in development against SARS-CoV-2, live attenuated virus (LAV) vaccines are a substantial minority despite the potential for nasal administration and the advantage of presenting all viral antigens to the host immune system[17–19]. To facilitate the evaluation of LAVs, we recently developed a genetic approach to attenuate SARS-CoV-2. Our strategy consists of three modifications to the viral genome: the removal of the furin cleavage site (PRRA)[20], the deletion of ORFs 6-8[21], and introduction of a pair of mutations to the Nsp1 gene (Fig. 1a)[22]. The resulted WA1-ΔPRRA-ΔORF6-8-Nsp1$^{K164A/H165A}$ (abbreviated as Nsp1-K164A/H165A in this paper) is attenuated both *in vitro* and *in vivo* compared to wildtype virus and was immunogenic and protective against the ancestral SARS-CoV-2 challenge[23]. In the current study, we assessed the mucosal immunogenicity, efficacy in protecting diseases caused by recent variants of concern, as well as the transmission of a candidate attenuated SARS-CoV-2 vaccine.

### Results

## Mucosal and systemic immunogenicity of Nsp1-K164A/H165A

First, we assessed anti-SARS-CoV-2 spike Immunoglobulin G (IgG) and IgA in serum samples and nasal washes from Syrian hamsters after intranasal inoculation of 100 PFU Nsp1-K164A/H165A or WA1/2020 SARS-CoV-2. Sera collected at 14- and 30-days post-infection (DPI) from both groups contained high titers of IgG antibodies specific for the WA1/2020 receptor binding domain (RBD) (Fig. 1b). Serum nAb titers increased from 0 to 14 DPI ($p < 0.0001$, mixed-effects analysis) and then from 14 to 30 DPI ($p < 0.0001$) for both groups of animals. Geometric mean titers (GMT) of serum nAbs against WA1/2020, indicated by 50% focus forming reduction neutralization titers ($FRNT_{50}$), were 2100 and 3169 at 30 DPI for Nsp1-K164A/H165A and WA1/2020 groups, respectively (Fig. 1c). At 30 DPI, IgA titers detected in nasal washes were significantly higher ($p < 0.0001$) in Nsp1-K164A/H165A (GMT 145) and WA1/2020 (GMT 68) groups compared to naive controls (Fig. 1d). In this experiment, there was 2-fold higher IgA titers ($p = 0.0336$) in the Nsp1-K164A/H165A group than in WA1/2020 infected animals. When the same sera were measured for anti-Delta variant RBD IgG, a 2-fold reduction was observed in both the WA1/2020 (GMT 139621 versus 250997) and Nsp1-K164A/H165A (GMT 127911 versus 243465) groups in comparison to WA1/2020 RBD-specific IgG titers (Fig. 1e in comparison to Fig. 1b). Concomitantly, serum nAb titers against Delta variant dropped by near 10-fold compared to those against the ancestral WA1/2020 for both Nsp1-K164A/H165A (GMT 215) and WA1/2020 (GMT 285) groups (Fig. 1f in comparison to Fig. 1c). Similarly, serum Omicron BA.1 RBD-specific IgG titers, in comparison to WA1/2020 RBD-specific IgG titers, decreased by nearly 4-fold in Nsp1-K164A/H165A-vaccinated animals (GMT 243465 versus 70613) and in WA1/2020-infected (GMT 250997 versus 62749) hamsters at 30 DPI (Fig. 1g compared to Fig. 1b). NAb against the BA.1 variant reached titers just above the limit of detection from both the Nsp1-K164A/H165A-vaccinated group (GMT 36, i.e., a > 50-fold reduction compared to nAb titers against the ancestral WA1/2020) and the WA1/2020-infected group (GMT 44.2, i.e, a > 70-fold reduction compared to nAb titers against the ancestral WA1/2020) (Fig. 1h in comparison to Fig. 1c).

To ensure the loss of serum neutralization antibody to Omicron BA.1 was not due to the low vaccine dose (100 PFU), we vaccinated Syrian hamsters with $10^4$ PFU Nsp1-K164A/H165A and characterized mucosal antibody response to and neutralization of Omicron subvariants. Because secretory IgA (SIgA) is critical to antiviral immunity in the lungs[24], we assessed anti-SARS-CoV-2 spike IgG and IgA in bronchoalveolar lavage fluid (BALF) and serum. At 14 and 28 DPI, serum anti-RBD IgG titers against WA1/2020 (GMT of 137772 and 163840, respectively) were 20-fold higher ($p < 0.0001$) than those against BA.1 variant (GMT of 3044 and 12177, respectively) (Fig. 2a). BALF collected on 14 and 28 DPI contained detectable levels of IgG specific for ancestral RBD (GMT of 452 and 761, respectively), however, IgG titers specific for BA.1 RBD were below the limit of detection (Fig. 2b). Serum WA1/2020 RBD-specific IgA titers decreased by 5.65-fold from 14 (GMT 115852) to 28 DPI (GMT 20480) ($p = 0.0233$, Sidak's multiple comparisons test), whereas serum anti-BA.1 RBD-specific IgA titers stayed at comparable levels at 14 and 28 DPI (Fig. 2c). In BALF, BA.1 RBD-specific IgA was undetectable at 14 and 28 DPI, but WA1/2020 RBD-specific IgA was detected at both 14 DPI ($p = 0.0003$) and 28 DPI ($p < 0.0001$) (Fig. 2d). NAb titers against WA1/2020 were 761 (GMT, 14 DPI) and 640 (GMT, 28 DPI) with great than 32-fold reductions when measured against Omicron subvariants BA.1, BA.2.12.1, BA.4, and BA.5 (Fig. 2e). Lastly, we assessed the cellular immunity elicited by Nsp1-K164A/H165A vaccination. Significant induction of IFNγ-secreting cells was observed at 14 DPI in splenocytes harvested from vaccinated group pulsed with nucleocapsid antigen pools N1 ($p = 0.0096$), N3 ($p = 0.0096$), and N4 ($p < 0.0001$) by ELISpot assays (Fig. 2f). Taken together, a single dose of Nsp1-K164A/H165A, when administered intranasally, induced IgA/IgG against SARS-CoV-2 spike protein in both respiratory tract and in circulation. However, these anti-Spike antibodies are variant-specific and subject to evasion by Omicron variants.

## Efficacy of Nsp1-K164A/H165A against challenge with Delta and Omicron variants

To assess whether intranasal administration of Nsp1-K164A/H165A offers protection against VOCs, male, 5-month-old Syrian hamsters were inoculated with 100 PFU of Nsp1-K164A/H165A (n = 14) or WA1/2020 (n = 14) by the intranasal route (derived from the study depicted in Fig. 1). At 35 days-post-infection (DPI), the vaccinated and convalescent hamsters (n = 7 per group) along with unvaccinated controls (n = 8 per group) were challenged with $10^4$ PFU of a Delta isolate (hCoV-19/USA/MD-HP05647/2021) or an BA.1 Omicron isolate (hCoV-19/USA/HI-CDC-4359259-001/2021). On 4 and 7 days-post-challenge (DPC), animals were euthanized to collect tissues for analysis of viral replication and pathology (Fig. 3a). Nasal wash samples were also collected from each hamster following challenge. We beheld a significant reduction of infectious virus in nasal wash samples at 2, 3, 4, and 5 DPC for the Delta challenged groups ($p < 0.0001$) inoculated with Nsp1-K164A/H165A or WA1/2020 compared to unvaccinated controls (Fig. 3b). Similarly, Nsp1-K164A/H165A vaccinated and WA1/2020 convalescent hamsters also exhibited reduced nasal viral load at 1 (WA1/2020, p = < 0.0001; Nsp1-K164A/H165A, p =

0.0001), 2 (WA1/2020 only, p = 0.0002), 3 (p < 0.0001), and 4 (p < 0.0001) DPC upon BA.1 challenge. By 5 DPC, infectious virus titers declined to baseline in all BA.1 challenged animals (Fig. 3c). At 4 DPC, envelope (E) subgenomic viral RNA (sgRNA), a hallmark of viral replication, was readily detected in the lungs, trachea, and nasal turbinates of Delta- and BA.1- challenged unvaccinated animals (Fig. 3d). By contrast, sgRNA levels were reduced by more than 4400-fold (p = < 0.0001) in the lungs, around 300-fold in trachea (p < 0.0001), and 300-fold in nasal turbinates (p < 0.0001) of Nsp1-K164A/H165A vaccinated and WA1/2020 convalescent animals after challenge with the Delta variant. As to BA.1 challenged animals, the reduction of sgRNA was greater than 100-fold in lungs (p < 0.0001), trachea (p = 0.0041), and nasal turbinates (p < 0.0001) at 4 DPC in both Nsp1-K164A/H165A vaccinated and WA1/2020 convalescent animals compared to the unvaccinated group. Infectious viral titers in lung homogenates also diminished below the limit of detection in Nsp1-K164A/H165A vaccinated and WA1/2020 convalescent groups following either Delta or Omicron BA.1 challenge at 4 DPC (Fig. 3e). At 7 DPC, sgRNA levels in the lungs were below the limit of detection except for the unvaccinated/challenged hamsters (Fig. 3f). Taken together, these results indicate that intranasal vaccination with Nsp1-K164A/H165A effectively reduces viral loads in both upper and lower respiratory tract of Syrian hamsters upon heterologous virus challenge.

To further examine the presence of viral antigens and host innate immune activation, lung sections from uninfected (mock) or challenged hamsters were stained with hematoxylin and eosin (H&E) and also immunostained for viral nucleocapsid protein (NP) and myxovirus resistance 1 (MX1), an interferon-induced antiviral host response marker[25,26] (Fig. 4). Lungs from Delta-challenged unvaccinated hamsters at 4 DPC showed widespread immune infiltrates and regions of viral NP deposition characterized by prominent staining of the epithelial lining of infected bronchioles accompanied by intense staining of surrounding alveolar epithelium (Fig. 4a&b). Two of the four BA.1-challenged unvaccinated animals at 4 DPC showed NP deposition with a similar staining pattern (Fig. 4b&c). Delta- and BA.1-challenged unvaccinated groups also showed increased MX1 immunoreactivity in these NP-positive lung regions particularly evident in the bronchiolar epithelium. Vaccination with Nsp1-K164A/H165A or infection with WA1/2020 blocked NP deposition and MX1 upregulation in the Delta- and BA.1-challenged groups (Fig. 4b&c). High resolution imaging of representative lung sections from Delta-challenged unvaccinated hamsters highlighted the pronounced upregulation of MX1 in nuclear and cytoplasmic compartments of infected bronchiolar epithelial cells and the attenuation in Nsp1-K164A/H165A-vaccinated hamsters (Fig. 4d). Altogether, the absence of NP staining and MX1 upregulation implies that the challenge virus, whether it is the Delta or Omicron BA.1 variant, failed to establish infection in the lungs of Nsp1-K164A/H165A-vaccinated and WA1/2020-convalescent hamsters.

Among the Delta-challenged hamsters, only the unvaccinated group (n = 8) significantly lost body weight over the course of 7 days (Fig. 5a). By contrast, none of the three groups lost weight after BA.1 challenge (Fig. 5a). At 4 DPC, percent of lung consolidation in Delta variant-challenged hamsters was significantly reduced in WA1/2020- (p = 0.0352, n = 3) and Nsp1-K164A/H165A- inoculated groups (p = 0.0386, n = 4) compared to unvaccinated controls (Fig. 5b). Accumulated pathology scores were also significantly lower at 4 DPC in Delta-challenged WA1/2020 ($p = 0.0002$) and Nsp1-K164A/H165A groups ($p < 0.0001$) as opposed to the unvaccinated- and Delta-challenged animals (Fig. 5b&c). Differing from the Delta variant, Omicron BA.1 challenge resulted in low pathology overall, with minimal consolidation (Fig. 5b) and low pathology scores at 4 DPC (Fig. 5c&d). At 7DPC, among Delta-challenged hamsters, both Nsp1-K164A/H165A and WA1/2020 inoculated groups (n = 3) had significantly ($p < 0.0001$) reduced consolidation compared to unvaccinated controls (Fig. 5e). Following BA.1 challenge, only two animals in the unvaccinated group had > 20% lung consolidation at 7 DPC (Fig. 5e), however, lung pathology was evident in 3 out of 4 animals within this group (Fig. 5f&g). In comparison, nearly no lung pathology was detected from Nsp1-K164A/H165A-vaccinated animals (n = 3, $p = 0.0195$).

To further characterize these histopathological changes using specific markers of inflammation and epithelial damage, serial lung sections from uninfected hamsters (mock), unvaccinated hamsters, and WA1/2020-convalescent and Nsp1-K164A/H165A-vaccinated hamsters were immunostained for Iba1 (a marker of macrophages), prosurfactant protein C (ProSPC, a marker of AT2 cells), RAGE (a marker of AT1 cells), and E-cadherin (a marker of intercellular epithelial junctions). At 7 DPC, lungs from all four Delta-challenged unvaccinated hamsters and two of the four BA.1-challenged unvaccinated hamsters showed regions of consolidation by H&E that corresponded with areas containing extensive accumulation of Iba1-expressing macrophages (Fig. 6a&b) and increased terminal deoxynucleotidyl transferase dUTP nick end labeling (TUNEL) (Fig. S1). These consolidated regions also showed marked reduction of ProSPC-expressing AT2 cells and pronounced loss of alveolar RAGE expression along the borders of AT1 cells. Distinct areas of epithelial cell loss and Iba1-positive macrophage consolidation formed around E-cadherin-labeled bronchioles that were presumably subject to virus attack (Fig. 6c). Increased E-cadherin staining also identified hyperplastic epithelium in consolidated spaces that was particularly prominent in Delta-challenged unvaccinated lungs. Vaccination with Nsp1-K164A/H165A attenuated Iba1-positive macrophage consolidation and protected against the loss of ProSPC and RAGE expression in Delta- and BA.1-challenge groups (Fig. 6a-c). Prior infection with WA1/2020 also protected against epithelial damage in the Delta-challenge group but, interestingly, appeared less effective in protecting against BA.1-induced inflammation and damage.

We then performed a correlational analysis between serum binding IgG, serum nAB, and nasal SIgA titers and tissue viral loads and severity of lung pathology. In general, antibody titers inversely correlated with tissue viral loads and severity of lung pathology, i.e., lower antibody titers correlate with increased frequencies of breakthrough infections (i.e., detectable viral loads) (Fig. S2). The exception is found in nasal turbinates following challenge with Omicron BA.1, where no clear correlation between antibody titers and viral loads was noticed. A plausible explanation of this finding is that the very low level or absence of BA.1-specific mucosal and systemic neutralizing antibodies failed to offer any protection in nasal turbinates (Fig. S2).

## Transmission of Nsp1-K164A/H165A in Syrian Hamsters

To characterize the transmissibility of Nsp1-K164A/H165A, we performed an additional study in an airborne transmission model (Fig. 7a) in which two Syrian hamsters in the same cage are separated from each other by a customized, perforated metal divider that prevents physical contact while permitting air exchange (*Movie. S1*)[27].

We first inoculated donor hamsters (male, 4-month-old, n = 14) with 100 PFU of WA1/2020 or Nsp1-K164A/H165A, which was previously shown to be immunogenic and protective against WA1/2020 challenge. Significant body weight loss was observed in WA1/2020 infected hamsters at 3–9 ($p < 0.0001$), 10–11 ($p < 0.001$), and 12–14 ($p < 0.01$) DPI and one had to be euthanized due to presentation of severe clinical signs (hypothermia, hunched posture, lethargy) at 7 DPI (Fig. 7b). By contrast, no significant weight loss was observed in Nsp1-K164A/H165A vaccinated animals (n = 14). WA1/2020 inoculated hamsters also exhibited significantly higher infectious viral loads in nasal wash samples at 1, 2, 3 DPI than Nsp1-K164A/H165A inoculated group (1 DPI, 158-fold, $p < 0.0001$; 2 DPI, ~ 33-fold, $p = 0.0003$; and 3 DPI, 10-fold, $p = 0.0105$) (Fig. 7c).

At 1 DPI, 7 sentinel hamsters were paired individually with either a WA1/2020 or a Nsp1-K164A/H165A inoculated hamster in cages with dividers. Nasal washes were collected from the sentinel hamsters from 1–4 days post-exposure (DPE) and seroconversion was determined after two weeks to confirm infection. Nasal wash infectious virus titers from Nsp1-K164A/H165A-exposed sentinels were undetectable at 1 DPE and then significantly lower at 2 DPE (> 100-fold, $p = 0.0006$) and 3 DPE (> 290-fold, $p < 0.0001$) compared to those from WA1/2020-exposed sentinels (Fig. 7d). At 4 DPE, there was no statistically significant difference between sentinel groups although 2 of the Nsp1-K164A/H165A sentinels had nasal wash titers below the detection limit (200 $TCID_{50}$/mL). Overall, the transmission of Nsp1-K164A/H165A to sentinel hamsters exhibited a delayed kinetics. No weight loss was evident in the sentinel hamsters exposed to Nsp1-K164A/H165A, however, WA1/2020-exposed sentinels experienced weight loss at 4–5 ($p < 0.05$), 6 ($p = 0.0005$), 7–12 ($p < 0.0001$), 13 ($p = 0.0003$), and 14 ($p = 0.0016$) DPE with a maximum mean weight loss of 15.6% on 9 DPE (Fig. 7e). By 14 DPE all exposed sentinel animals had high levels of anti-RBD IgG in serum (Fig. 7f) except one animal from the Nsp1-K164A/H165A-exposed sentinel group, which did not seroconvert (there was also no detectable infectious virus in nasal washes collected from this animal).

At 4.5 months after exposure (MPE), all seroconverted sentinel animals were tested for nAb titers against WA1/2020 and BA.2.12.1. Serum nAb titers against WA1/2020 from WA1/2020 exposed sentinel animals (GMT 4637) were 4-fold higher ($p = 0.0026$, unpaired t-test) than Nsp1-K164A/H165A-exposed sentinel hamsters (GMT 1140) (Fig. 7g). Neutralization of BA.2.12.1, however, was observed in only 2 animals from the Nsp1-K164A/H165A sentinel group and 5 animals from the WA1/2020 sentinel group prior to challenge (Fig. 7h).

## Passively immunized sentinel hamsters are protected from BA.2.12.1 challenge

Four and half months after the initial exposure, seroconverted sentinel hamsters from Fig. 7 were challenged with $10^4$ FFU Omicron BA.2.12.1 by the intranasal route. Weight loss did not occur in any

BA.2.12.1 challenged hamsters (Fig. 8a). Reduced nasal viral loads in WA1/2020-exposed sentinel hamsters (n = 7) were noticed on days 3 (p = 0.0492) and 4 (p = 0.0.0011) DPC compared to naïve hamsters that were challenged with BA.2.12.1 (Fig. 8b). Interestingly, despite low or absent BA.2.12.1-specific nAB titers in Nsp1-K164A/H165A sentinel animals (Fig. 7h), nasal viral load of these animals was significantly lower compared to controls at 4 DPC (p = 0.0094) (Fig. 8b). Infectious virus titers in BALF were at least one-log lower in WA1/2020 and Nsp1-K164A/H165A sentinel hamsters than in naïve controls at 4 DPC (p < 0.0001) (Fig. 8c). Infectious virus titers in lung homogenates of the BA.2.12.1 infected control animals (n = 4) were around 1000 FFU/mL at 4 DPC, whereas 2 out 4 sentinel hamsters had no detectable infectious virus in the lungs (Fig. 8d). Consequently, $log_{10}$-transformed sgRNA copies detected in both Nsp1-K164A/H165A (p = 0.0043) and WA1/2020 sentinel hamster lungs (p = 0.0004) were at least one order of magnitude lower compared to controls at 4 DPC (Fig. 8e).

Histopathology analyses of fixed lungs revealed minimal consolidation at 4 DPC (n = 4) and 7 DPC (n = 3) in all groups. A slightly higher percentage of lung consolidation (Fig. 8f) was observed in WA1/2020-exposed sentinels compared to Nsp1-K164A/H165A (4 DPC, p = 0.0490) or Nsp1-K164A/H165A sentinel hamsters (p = 0.0269) and control animals (p = 0.0326) at 7 DPC. The consolidation is most likely remnant pathology from the initial WA1/2020 infection, but not due to BA.2.12.1 challenge. There was no significant difference in pathology scores at 4 or 7 DPC between groups (Fig. 8g), although WA1/2020-exposed sentinel hamsters did show bronchiole mucosal hyperplasia at 4 DPC (Fig. 8h) and 7 DPC (Fig. 8i). Noticeable lung pathologies in naive animals after BA.2.12.1 challenge included alveolar wall thickening, airway infiltrates, and type II pneumocyte hyperplasia. By contrast, such pathologies were absent in the lungs of Nsp1-K164A/H165A exposed sentinel hamsters after challenge (Fig. 8h&i). Altogether, Nsp1-K164A/H165A exposed sentinel hamsters were protected from BA.2.12.1 challenge in the lungs.

## Discussion

Current SARS-CoV-2 vaccines, particularly the mRNA-based ones, induce robust systemic humoral and cellular immunity and prevent severe disease caused by SARS-CoV-2[28]. However, protection against infection and transmission of SARS-CoV-2 variant viruses, in particular the Omicron sub-lineage viruses, by mRNA vaccines may be limited[29–31]. Results from recent studies suggest that intramuscular vaccination tends to induce potent systemic but not local humoral response at the mucosa. By contrast, nasal vaccines induce more robust mucosal immune responses, characterized by secretion of IgA and IgG at mucosal surfaces and detection of resident memory T/B cells in respiratory tract[16,24,32–34]. Here we show that intranasal immunization of Syrian hamsters with an attenuated SARS-CoV-2 (Nsp1-K164A/H165A) induced SIgA and IgG at respiratory mucosa, albeit the levels of mucosal IgA/IgG appeared to be at least two orders of magnitude lower than their serum counterparts. Vaccinated hamsters were largely protected in the lungs from subsequent challenge with Delta, Omicron BA.1 and BA.2.12.1, even when circulating nAb titers against the latter two variants were below limit of quantification. Together, our study demonstrates the great potential of attenuated Nsp1-K164A/H165A as a nasal vaccine capable of inducing SIgA/IgG in the respiratory mucosa.

Many of the pathologies found in human cases of COVID-19 are recapitulated in mature (> 4-6-month-old) Syrian hamsters, making them an excellent animal model for studying SARS-CoV-2 pathogenesis[35]. For example, the damage to AT1 and AT2 cells with the prominent infiltration of macrophages observed in the lungs of Delta- and BA.1-challenged unvaccinated hamsters partially mimics the histopathology of COVID-19 in humans[36,37]. One of the challenging aspects of this model, however, is the relative lack of species-specific reagents that has made immunological analyses in Syrian hamsters difficult. In this study we established methods to reliably measure SIgA/IgG in nasal wash and BALF samples. These techniques have now enabled us to assess the mucosal immunogenicity of SARS-CoV-2 vaccine candidates in Syrian hamsters. We detected significant amounts of SIgA/IgG in nasal wash and BALF samples from hamsters after intranasal vaccination of Nsp1-K164A/H165A. One significant and noteworthy observation is that SIgA/IgG titers, like circulating nAb titers, inversely correlate with viral loads in respiratory tract and the severity of lung pathology (Fig. S2). When SIgA/IgG is specifically raised against the ancestral virus (WA1/2020) and hence does not efficiently neutralize Omicron subvariants, vaccinated hamsters were not free of virus in the upper respiratory tract upon challenge with Omicron sub-lineage viruses. This finding is in line with the recent observation that anti-Spike mucosal IgA protects against SARS-CoV-2 Omicron infection in human population[38]. Interestingly, Nsp1-K164A/H165A-vaccinated animals were protected in the lung against both Delta and Omicron BA.1 challenge. It is possible that cellular immunity, induced by Nsp1-K164A/H165A, contributes to the protection against heterologous virus challenge, as we detected splenocytes reacting to the nucleocapsid protein of the virus. Future research is warranted to decipher the mechanism of live attenuated viral vaccines in conferring cross-protection in hamsters as more reagents become available.

An early and robust activation of interferon (IFN) signaling pathways contributes to the protective mucosal immune response against viral infections in the respiratory tract[39]. Viral recognition in infected immune and/or epithelial cells triggers the production of IFNs (types I, II, and III) that subsequently limit viral replication and dissemination by activating the transcription of numerous antiviral IFN-stimulated genes (ISGs) including IFIT1-3, OAS1, IRF7 and MX1 in infected cells and bystander cells. While IFNs are critical to the antiviral host defenses, excessive or persistent IFN signaling may also aggravate lung pathologies of viral infections including SARS-CoV-2[40]. Activation of IFN signaling in the lungs of Delta- and BA.1 unvaccinated animals was clearly evidenced by the robust upregulation of MX1 protein that localized specifically to infected bronchioles and neighboring alveolar parenchyma stained by viral NP. In this regard, we surmise that the lack of MX1 upregulation in the lungs of Nsp1-K164A/H165A-vaccinated hamsters supports that the vaccine exerts a strong and specific protective response along the upper airways that blocks viral dissemination in the lungs. The lack of macrophage accumulation in the lungs of Nsp1-K164A/H165A-vaccinated hamsters suggests that robust viral infection did not occur, hence vaccinated animals did not develop pneumonia.

An intrinsic safety concern for a LAV against a pandemic virus is the potential transmission of the vaccine virus. In principle, transmission of a vaccine virus may contribute to the establishment of herd immunity, but there is a possibility that an attenuated virus may directly or through reversion to a more virulent form cause illness among some at-risk individuals. We have previously demonstrated the genome stability of Nsp1-K164A/H165A[23]. Here we found that the airborne transmission of Nsp1-K164A/H165A was less efficient compared to wild-type SARS-CoV-2 in hamsters, but most (except for one animal) sentinel hamsters became seroconverted after 14 days. Airborne transmission of Nsp1-K164A/H165A did not cause weight loss in sentinel hamsters. Another intriguing finding is that four and half months after the initial exposure, these seroconverted hamsters were largely protected from a BA.2.12.1 challenge. Thus, passive immunization via transmission of Nsp1-K164A/H165A was achieved. Nonetheless, the risks and benefits of administering an infectious attenuated SARS-CoV-2 vaccine will need to be further evaluated.

## Study Limitations

Typically, SARS-CoV-2 infection of Syrian hamsters induces pronounced consolidation and pathologies in the lung at 7 DPC[35]. However, lung pathology was limited to a subset of BA.1 and BA.2.12.1-infected hamsters rather than being widespread as in Delta challenged animals. For this reason, determination of vaccine efficacy against Omicron sub-lineage viruses using Syrian hamsters was problematic due to the low pathogenicity of BA.1 and BA.2.12.1 in this species. Low pathology scores in unvaccinated controls led to difficulties in determining efficacy of vaccination in preventing lung damage post-challenge. Additionally, persistent damage after WA1/2020 infection likely led to increases in bronchiole mucosal hyperplasia in hamsters observed at 4 DPC in convalescent animals challenged with Delta as well as BA.1 and BA.2.12.1 Omicron. In separate experiments being prepared for publication, we have observed long-term lung damage (including hyperplasia in bronchioles) in hamsters up to 4 weeks after WA1/2020 infection by the intranasal route. This would provide an explanation for the observed lung pathology in WA1/2020 convalescent hamsters after Omicron challenge which was not observed in control or Nsp1-K164A/H165A vaccinated animals.

### Concluding Remarks

Nsp1-K164A/H165A vaccination induces humoral immunity at mucosa as well as cellular-immunity targeting nucleocapsid protein. Additionally, transmission of Nsp1-K164A/H165A leads to passive immunization. Thus, Nsp1-K164A/H165A may be further developed into a nasal vaccine for primary series or as a booster.

### Methods

## Cells and Viruses

Vero E6 cell line (Cat # CRL-1586) was purchased from American Type Culture Collection (ATCC) and cultured in Dulbecco’s minimal essential medium (MEM) supplemented with 10% fetal bovine serum

(Invitrogen) and 1% penicillin/streptomycin and L-glutamine. Calu-3 cell line (Cat # HTB-55) was obtained from ATCC and maintained in EMEM + 20%FBS. H1299-hACE2 is a human lung carcinoma cell line stably expressing human ACE2. It was made by S. Liu in our group from the NCI-1299 human lung carcinoma cell line (ATCC CRL-5803). H1299-hACE2 cells were maintained in DMEM supplemented with 5% penicillin and streptomycin, and 10% fetal bovine serum (FBS) at 37°C with 5% CO2.

Production of Nsp1-K164A/H165A was described elsewhere[23]. The SARS-CoV-2 isolate WA1/2020 (NR-52281, lot 70033175) was obtained from BEI Resources, NIAID, NIH, and had been passed three times on Vero cells and 1 time on Vero E6 cells prior to acquisition. It was further passed once on Vero E6 cells in our lab. SARS-CoV-2 hCoV-19/USA/MD-HP05647/2021 (Delta variant, Pango lineage B.1.617.2) was obtained from BEI resources, NIAID, NIH (NR-55672, Lot 70046635) and had been passaged once in Vero E6-TMPRSS2 and once in Calu-3 cells prior to acquisition. It was passaged once more in H1299-hACE2 cells in our lab to generate viral stocks. Passaged viruses were deep sequenced to confirm identity. SARS-CoV-2 isolates hCoV-19/USA/HI-CDC-4359259-001/2021 (B.1.1.529 Omicron, NR-56475), hCoV-19/USA/NY-MSHSPSP-PV56475/2022 (BA.2.12.1 Omicron, NR-56782), USA/MD-HP30386/2022 (BA.4, Omicron, NR-56802), and hCoV-19/USA/COR-22-063113/2022 (BA.5 Omicron, NR-58620) were obtained from BEI resources and used directly in experiments.

## Hamster Challenge Experiments

Adult male and female outbred Syrian hamsters were previously purchased from Envigo and held at FDA vivarium. All experiments were performed within the biosafety level 3 (BSL-3) suite on the White Oak campus of the U.S. Food and Drug Administration. The animals were implanted subcutaneously with IPTT-300 transponders (BMDS), randomized, and housed 2 per cage in sealed, individually ventilated rat cages (Allentown). Hamsters were fed irradiated 5P76 (Lab Diet) *ad lib*, housed on autoclaved aspen chip bedding with reverse osmosis-treated water provided in bottles, and all animals were acclimatized at the BSL3 facility for 4–6 days or more prior to the experiments. The study protocol details were approved by the White Oak Consolidated Animal Care and Use Committee and carried out in accordance with the PHS Policy on Humane Care & Use of Laboratory Animals.

Adult male (5–6 months old) Syrian hamsters (*Mesocricetus auratus*) were anesthetized with (3–4% v/v) isoflurane and oxygen following procedures as described previously [35,41,42]. Intranasal inoculation was done by pipetting $10^2$ PFU or $10^4$ PFU SARS-CoV-2 in 50 µl volume dropwise into the nostrils of the hamster under anesthesia. Following infection, hamsters were monitored daily for clinical signs and weight loss. Nasal washes were collected by pipetting ~ 200 µl sterile phosphate buffered saline into one nostril when hamsters were anesthetized by 3–5% isoflurane. Nasal swabs were done as described previously [32].

For airborne transmission, a subset (n = 7) of hamsters inoculated with $10^2$ PFU WA1/2020 or Nsp1-K164A/H165A were paired in divided cages to prevent direct contact to measure transmission to naive sentinels [27]. One hamster (WH363), paired with an actively shedding Nsp1-K164A/H165A vaccinated animal, did not show evidence of productive infection or seroconvert at 14 DPE and remained seronegative until just prior to BA.2.12.1 challenge 4.5 months later. For these reasons, WH363 was removed from the challenge datasets.

For tissue collection, a subset of hamsters was humanely euthanized by intraperitoneal injection of pentobarbital at 200mg/kg at 4 and 7 DPC. Lungs, trachea, and nasal turbinates were dissected for histopathology or homogenized for RNA extraction or titration in cell culture. Blood collection was performed under anesthesia (3–5% isoflurane) through gingival vein puncture or cardiac puncture when animals were euthanized. The left lobes of hamster lungs (~ 0.2 gram) were diced, divided, and resuspended in 1 milliliter MEM or TriZol reagent (RNA extraction) and homogenized on a Precellys Evolution tissue homogenizer with a Cooling Unit (Bertin). Trachea and nasal turbinates were homogenized the same way in TriZol Reagent. Splenocytes were extracted at 14 DPI from vaccinated and naive hamsters and IFNγ-secreting cells were identified after stimulation with spike and nucleocapsid antigen pools (Genscript) by ELISpot (MABTECH, 3102-2H).

## RNA isolation and qRT-PCR

Procedures as described previously[35,41]. In brief, RNA was extracted from 0.1-gram tissue homogenates using QIAamp vRNA mini kit or the RNeasy 96 kit (QIAGEN) and eluted with 60 µl of water. 5 µL RNA was used for each reaction in real-time RT-PCR. When graphing the results in Prism 9, values below the limit of quantification (LoD) were arbitrarily set to half of the LoD values. Unless otherwise specified, the unit for RNA copies are as presented as $Log_{10}$ RNA copes/µg tissue RNA.

## Histopathology Analyses

Procedures as described previously[35,41]. Tissues (lungs, trachea, and nasal turbinates) were fixed in 10% neutral buffered formalin overnight and then processed for paraffin embedding. The 5-µm sections were stained with hematoxylin and eosin for histopathological examinations. Images were scanned using an Aperio ImageScope. Blinded samples were graded by a licensed pathologist for the following twelve categories: consolidation, alveolar wall thickening, alveolar airway infiltrates, perivascular infiltrates, perivascular edema, type II pneumocyte hyperplasia, atypical pneumocyte hyperplasia, bronchiole mucosal hyperplasia, bronchiole airway infiltrates, proteinaceous fluid, hemorrhage, and vasculitis. Grading: 0 = none, 1 = mild, 2 = moderate, 3 = severe. A graph was prepared by summing up the score in each category.

## Virus titration

Tissue culture infectious dose 50% ($TCID_{50}$) assays were done described previously [35,41] for initial nasal wash titrations post-inoculation. In brief, Vero E6 cells were plated the day before infection into 96 well plates at $1.5 \times 10^4$ cells/well. On the day of the experiment, serial dilutions of 20 µl nasal wash samples were made in media and a total of six to eight wells were infected with each serial dilution of the virus. After 48 h incubation, cells were fixed in 4% PFA followed by staining with 0.1% crystal violet. The TCID50 was then calculated using the formula: log (TCID50) = log(do) + log (R) (f + 1). Where do represents the dilution giving a positive well, f is a number derived from the number of positive wells calculated by a moving average, and R is the dilution factor.

For focus-forming assay, nasal wash, BALF, and lung homogenate samples were 10-fold serially diluted in 96-well plates and dilutions added to 96-well black-well plates for fluorescent focus forming assays in H1299-hACE2 cells[43]. After 1 h the Tragacanth gum overlay (final concentration 0.3%) was added. Cells were incubated at 37°C and 5% $CO_2$ for 1 day, then fixed with 4% paraformaldehyde, followed by staining of cells with primary rabbit anti-N Wuhan-1 antibody (Genscript) overnight followed by secondary anti-rabbit Alexa-488 conjugated antibody and DAPI staining. The infectious titers were then counted using Gen5 software on a Cytation7 machine and calculated and plotted as focus forming units per milliliter (FFU/ml).

## SARS-CoV-2 neutralization assay

Samples were serially diluted 2-fold in 5% FBS DMEM and mixed with 100 PFU of SARS-CoV-2 in a 96-well plate at 37°C for 1 hour. Sample:virus mixtures were then added to confluent H1299-hACE2 cells in 96-well plates. Cells were infected for 1 hour before the inoculum was removed and washed three times with DPBS. A second overlay containing 1.2% Tragacanth gum, 2X MEM, 5% FBS, and DMEM was added to the plate. Cells were incubated at 37°C for 1 day, then fixed with 4% paraformaldehyde, followed by staining of cells with primary rabbit anti-SARS-CoV-2 N antibody (Genscript U739BGB150-5) overnight followed by secondary anti-rabbit Alexa-488 conjugated antibody and 4',6-diamidino-2-phenylindole (DAPI) staining. Plates were imaged on a Cytation7 (Agilent), and foci were counted using Gen5 software. For the neutralization assays, recombinant LY-CoV555 (Bamlanivimab) mixed with WA1/2020 [44] was included as a positive control. The 50% endpoint neutralization titers were determined as the reciprocal of the highest dilution providing ≤ half of the number of foci obtained from the negative control well (plain DMEM mixed with 100 PFU virus).

## Measurement of antibody by ELISA

The preparation of SARS-CoV-2 RBD antigen in a baculovirus expression system and its use in ELISA were previously described[45]. ELISAs were performed with slight modifications. Briefly, Immulon $2^{HB}$ plates were coated with recombinant RBD protein at 1 µg/mL overnight at 4°C. Test serum samples were pre-diluted in assay diluent (PBS containing 0.05% Tween-20 [PBST] and 10% fetal bovine serum), followed by serial two-fold dilutions of each sample in duplicates across the plate. A starting dilution of 1:160, 1:80, and 1:20 was used for serum (IgA and IgG), BALF (IgG) and nasal wash and BALF (IgA) samples, respectively. Plates were incubated with the test serum samples for 2 h at 37°C. After rigorous plate washes in a microplate washer, plates were incubated with anti-hamster antibodies. For IgG ELISA, a 1:4000 dilution of an HRP-conjugated goat anti-hamster IgG (6060-05, Southern Biotech, Birmingham, Alabama) was added to assay wells. For IgA ELISA, a rabbit anti-hamster IgA antibody [sandwich antibody; (cat. #sab 3001a) Brookwood Biomedical, Jemison, Alabama] was added to assay wells at 1:4000 dilution and plates incubated for 1 hour at 37°C. Unbound sandwich antibody was washed off and a 1:4000 dilution of an HRP-conjugated goat anti-rabbit IgG (4030-05, Southern Biotech, Birmingham,

Alabama) was added to assay plates. In both IgG and IgA ELISAs, incubation with HRP-conjugated secondary antibodies lasted 1 hour after which plates were rigorously washed to remove unbound antibodies. The ABTS/$H_2O_2$ peroxidase substrate (SeraCare, Gaithersburg, Maryland) was added to assay wells and plates left at room temperature for 20 to 30 minutes. Color development was stopped by adding 1% SDS and $OD_{405}$ values were captured on the VersaMax microplate reader with Softmax Pro 7 software (Molecular Devices). In the IgG ELISA, the mean $OD_{405}$ values of PBS treatment groups were subtracted from the mean $OD_{405}$ values from other treatment groups and the assay endpoint was a mean $OD_{405}$ value 0.05 (i.e., after background subtraction). In the IgA ELISA, the assay endpoint was a mean $OD_{405}$ value 0.02 of duplicate wells. Antibody titer was defined as the reciprocal of the highest dilution of a sample at which the mean $OD_{405}$ value for duplicate wells was 0.02 (IgA) or 0.05 after background subtraction (IgG).

## IFN-gamma ELISpot

Hamster interferon gamma (IFN-γ) enzyme-linked immunosorbent spot (ELISpot) analysis was performed using the Hamster IFN-γ ELISpotBASIC (MABTECH Mabtech 3102-2H, Nacka Strand, Sweden) kit according to the manufacturer's instructions. MSIP Plates (Millipore) were washed 5 times with sterile water, coated with mAb (MTH21) and incubated overnight at 4°C. Coated plates were washed 5 times with 1X PBS, blocked for 30 minutes (room temperatures) with supplemented RPMI 1640 (GibcoBRL) containing 10% heat inactivated FBS, 1% 100x penicillin, streptomycin, and L-Glutamine solution (GibcoBRL). $2.5x10^5$ freshly isolated splenocytes were seeded in each well and stimulated for 45–48 hours at 37°C with SARS CoV-2 Spike proteins peptide pools (2 μg/ml each peptide) (BEI Catalog No. NR-52418) or Nucleocapsid proteins peptide pools (2 μg/ml each peptide) (BEI Catalog No. NR-52419) prepared in serum free RPMI 1640. Negative and positive plate controls were medium or 2μg/ml concanavalin A (ConA, Sigma-Aldrich), respectively. Plates were incubated with 1μg/ml mAb (MTH29-biotin) for 2 hours, and then 1 hour with Streptavidin-HRP, and finally developed after adding TMB substrate (product No. 3651-10). Distinct spots typically emerge within 20 minutes. After drying, spots were counted using a BioTek Cytation 7 imaging reader (Agilent) and analysis software Gen5 Version No. 3.11. ELISpot data was analyzed in Microsoft excel. The average number of spots from two negative wells (unstimulated cells) was subtracted from peptide pools stimulated wells for each plate. Results were expressed as difference in spots forming cells (SFC)/$10^6$ PBMC between negative control and peptide pools stimulations conditions. Results were plotted using GraphPad Prism 9.

## Lung immunofluorescence analyses

Formalin-fixed paraffin-embedded (FFPE) lung sections 4 μm thick were dewaxed, rehydrated, and heat-treated in a microwave oven for 15 minutes in 10 mM Tris/1 mM EDTA buffer (pH 9.0). After cooling for 30 min at room temperature, heat-retrieved sections were blocked in PBST with 2.5% bovine serum albumin (BSA) for 30 min at RT followed by overnight incubation at 4°C with primary antibodies in 1% BSA. Primary antibodies used included SARS nucleocapsid protein (NP) (1:800, Sino Biologicals, 40143-MM05), MX1 (Proteintech, 13750-1-AP), prosurfactant protein C (ProSPC) (1:200, EMD Millipore, AB3786),

Iba1 (1:100, Abcam, ab5076), RAGE (1:400, Abcam, ab216329), and E-cadherin (ECAD) (Abcam, ab219332). Sections were rinsed and incubated with Alexa Fluor 488 (A-21206) and Alexa Fluor 647-conjugated secondary antibodies (A-31571, A-21447) for 1 hour at RT (ThermoFisher, Waltham, MA). Nuclei were counterstained with Hoechst 33342. For double labeling experiments, primary antibodies were mixed and incubated overnight at 4°C. For negative controls, sections were incubated without the primary antibody or mouse and rabbit isotype antibody controls. Sections stained with conjugated secondary antibodies alone showed no specific staining. Whole slide fluorescence imaging was performed using a Hamamatsu NanoZoomer 2.0-RS whole-slide digital scanner equipped with a 20x objective and a fluorescence module #L11600. Analysis software NDP.view2 was used for image processing (Hamamatsu Photonics, Japan). Immunofluorescence and differential interference images were also captured using an Axio Observer Z1 inverted microscope (Carl Zeiss, Thornwood, NY) equipped with an Axiocam 506 monochrome camera, an ApoTome.2 optical sectioning system, and a Plan-Apochromat 63x/1.4NA oil immersion with WD = 0.19 and Plan-Apochromat 20x/0.8 objective lens. Digital image post-processing and analysis were performed using the ZEN 2 ver. 2.0 imaging software. Images were constructed from Z-stack slices collected at 0.48 μm intervals (5 μm thickness in total) and visualized as maximum intensity projections in orthogonal mode. For semiquantitative analysis of NP staining, high resolution whole-slide digital images of each lung section were acquired and the NDP.view2 software was used to measure the NP-stained area as a percentage of total area of the section. For TUNEL staining, sections were deparaffinized, hydrated, and pretreated with Proteinase K, followed by EDTA, distilled H O wash, and BSA blocking. Sections were then incubated in a reaction mixture (TdT, dUTP, and buffer), washed, and incubated with anti-digoxigenin antibody. Sections were then visualized with alkaline phosphatase-ImmPACT Vector Red and counterstained with hematoxylin.

### Statistical analysis

One-way ANOVA or Student t-test was used to calculate statistical significance through GraphPad Prism (9.1.2) software for Windows, GraphPad Software, San Diego, California USA, www.graphpad.com.

## DATA and MATERIALS AVAILABILITY

All data are available in the main text or the supplementary materials. All unique/stable reagents generated in this study are available from the Lead Contact with a completed Materials Transfer Agreement.

## Declarations

### Acknowledgments

We would like to thank the White Oak FDA Animal Program Staff and Veterinarians: John Dennis, Mario Hernandez, Mario Ortega, and Eric Nimako. The following reagents were obtained through Biodefense and Emerging Infections Research Resources Repository, NIAID, NIH: SARS-Related Coronavirus 2, Isolate USA-WA1/2020, NR-52281; Isolate hCoV-19/USA/MD-HP05647/2021 (Lineage B.1.617.2; Delta variant),

NR-55672, contributed by Dr. Andrew S. Pekosz; Isolate hCoV-19/USA/HI-CDC-4359259-001/2021 (Lineage B.1.1.529; Omicron Variant, BA.1), NR-56475, contributed by Centers for Disease Control; Isolate hCoV-19/USA/NY-MSHSPSP-PV56475/2022 (Lineage BA.2.12.1; Omicron Variant), NR-56781, deposited by Dr. Viviana Simon; Isolate hCoV-19/USA/COR-22-063113/2022 (Lineage BA.5; Omicron Variant), NR-58616, contributed by Dr. Richard J. Webby. We thank Drs. A. Rosenfeld and A. Kachko for critical reading the manuscript.

**Funding:** The work described in this manuscript was supported by U.S. Food and Drug Administration (FDA) intramural research fund. K. S. was supported by the U.S. FDA, Office of the Chief Scientist, Medical Countermeasures Initiative, under OCET Intramural Program ID# 2022-1821. The funders had no role in study design, data collection and analysis, decision to publish, or preparation of the manuscript. The content of this publication does not necessarily reflect the views or policies of the Department of Health and Human Services, nor does mention of trade names, commercial products, or organizations imply endorsement by the US Government.

**Author contributions:**

Conceptualization: C.B.S., T.T.W.

Methodology: C.B.S., F.D., P.S., C.A.M., S.L., T.T.W.

Investigation: C.B.S., P.S., F.D., C.A.M., C.L.P., S.L., K.S., C.Z.L., M.F.S., J.P.W., T.T.W.

Visualization: C.B.S., F.D., T.T.W.

Funding acquisition: F.D., T.T.W.

Project administration: T.T.W.

Supervision: J.P.W., T.T.W.

Writing: C.B.S., F.D., T.T.W.

**Competing Interests:** C.B.S., P.S., S.L., C.Z.L. and TTW are inventors on a patent filed by the U.S. FDA based on the results described in this manuscript.

## Figures

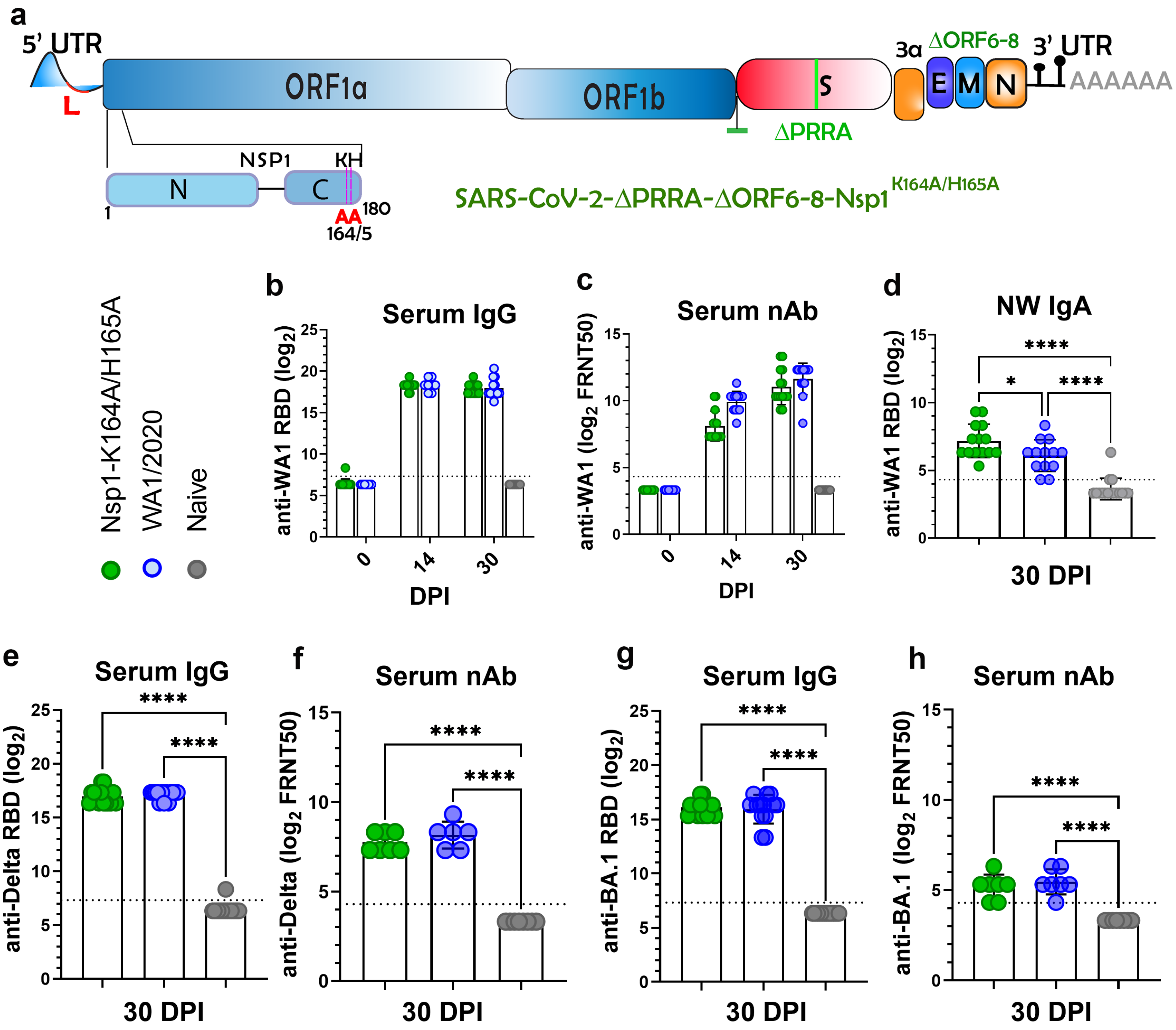

**Figure 1**

**Intranasal immunization with 100 pfu Nsp1-K164A/H165A induces IgG and IgA**. **a** Genome organization of the attenuated SARS-CoV-2-DPRRA-DORF6-8-Nsp1$^{K164A/H165A}$ (abbreviated as Nsp1-K164A/H165A). The polybasic insert "PRRA" together with ORF6-8(green) were removed from the WA1/2020 genome. Locations of K164A/H165A within Nsp1 are indicated at the bottom left of the panel. **b to h** Hamster sera or nasal wash samples were collected at 14- and 30-days post-intranasal inoculation of 100 PFU Nsp1-K164A/H165A or WA1/2020 and then tested for binding to WA1-2020 receptor binding domain (RBD) by ELISA (**b**) and for neutralization against WA1/2020 (**c**). Samples from naïve hamsters were included as negative controls. **d** Secretory IgA levels in nasal wash samples (NW IgA) collected 30 DPI were measured by ELISA (n=14). **e-h** Hamster sera of 30 DPI were measured for anti-Delta RBD IgG (**e**) and neutralization (**f**), for anti-Omicron BA.1 RBD IgG (**g**) and neutralization (**h**). Bar graphs indicate mean titers with standard deviations shown as error bars. Each solid circle indicates individual hamsters from a single experiment. Statistical differences were calculated using ordinary one-way analysis of variance (ANOVA) in GraphPad Prism 9.4.0 with Tukey's multiple comparisons tests. For statistical significance, * indicates p<0.05 and **** indicates p<0.0001. DPI= days post-infection.

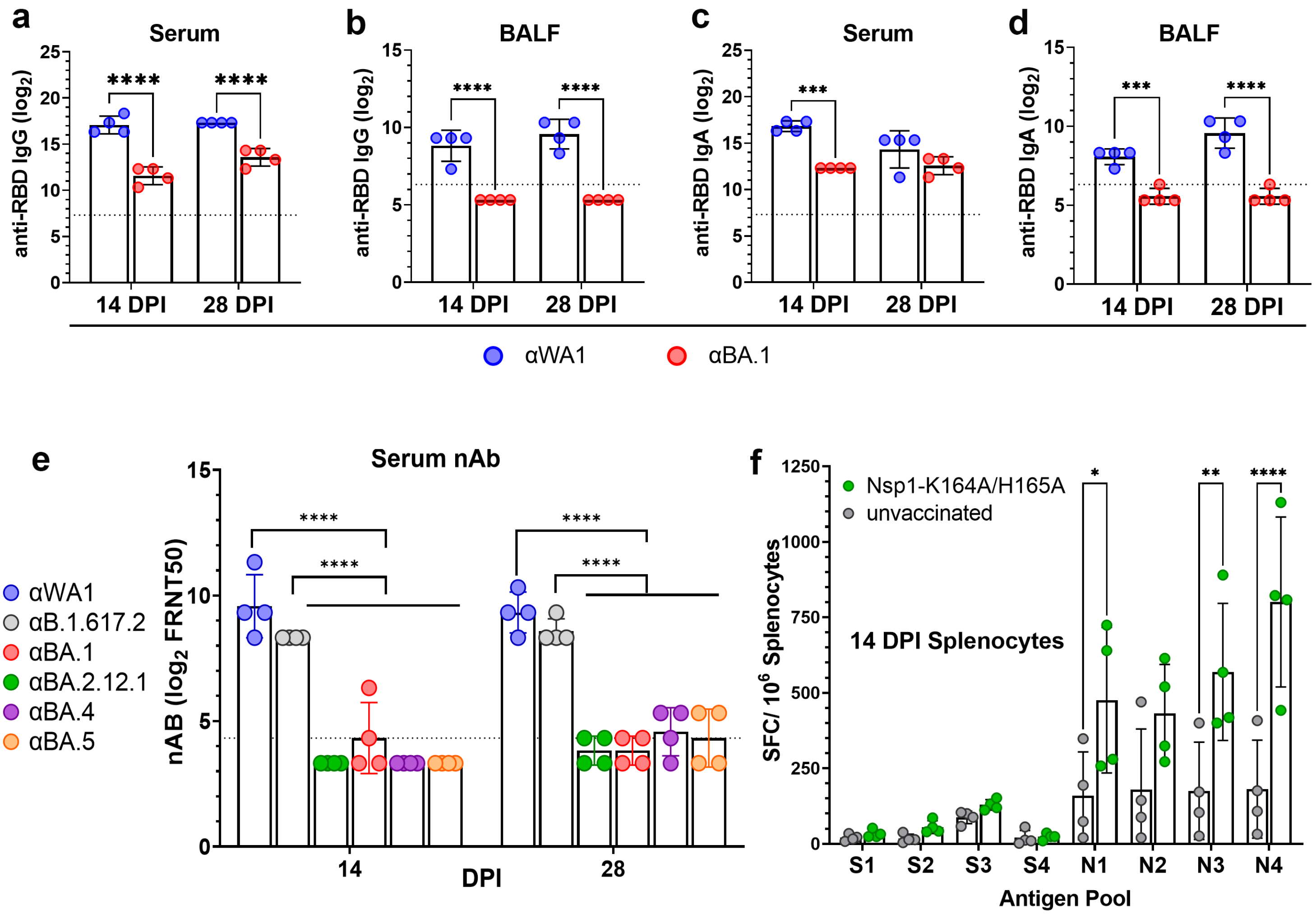

### Figure 2

**Intranasal immunization with Nsp1-K164A/H165A induces mucosal and systemic humoral immunity and cellular immunity.** Male (4-month-old, n=8) Syrian hamsters were intranasally vaccinated with $10^4$ PFU Nsp1-K164A/H165A. Animals were bled at 14 and 28 DPI to collect sera and n=4 animals were euthanized at each time point to collect broncho-alveolar lavage fluid (BALF). Anti-RBD IgG titers against WA1/2020 (blue) or BA.1 (red) in serum (**a**) and BALF (**b**) were measured by ELISA. Anti-RBD IgA was likewise quantified in serum (**c**) and BALF (**d**). **e** Serum nAb titers at 28 DPI were measured against WA1/2020 (WA1), Delta (B.1.617.2), as well as Omicron subvariants (BA.1, BA.2.12.1, BA.4, BA.5) using a 50% focus reduction neutralization (FRNT50) assay. **f** Splenocytes were isolated from naive and vaccinated hamsters at 14 DPI and pulsed with 10 mM WA1/2020 Spike (S) and Nucleocapsid (N) antigen pools for 48 hours. IFNg-secreting splenocytes were enumerated by ELISPOT. Bar graphs in panels **a-e** represent samples collected at two time points from the same animals in a single experiment with dots representing individual animals. Statistical differences were calculated using ordinary one-way analysis of variance (ANOVA) in GraphPad Prism 9.4.0 with Tukey's multiple comparisons tests. ELISPOT data were compared using two-way ANOVA with Sidak's multiple comparison test. For statistical significance, * indicates p<0.05, ** indicates p<0.01, and **** indicates p<0.0001. DPI= days post-infection.

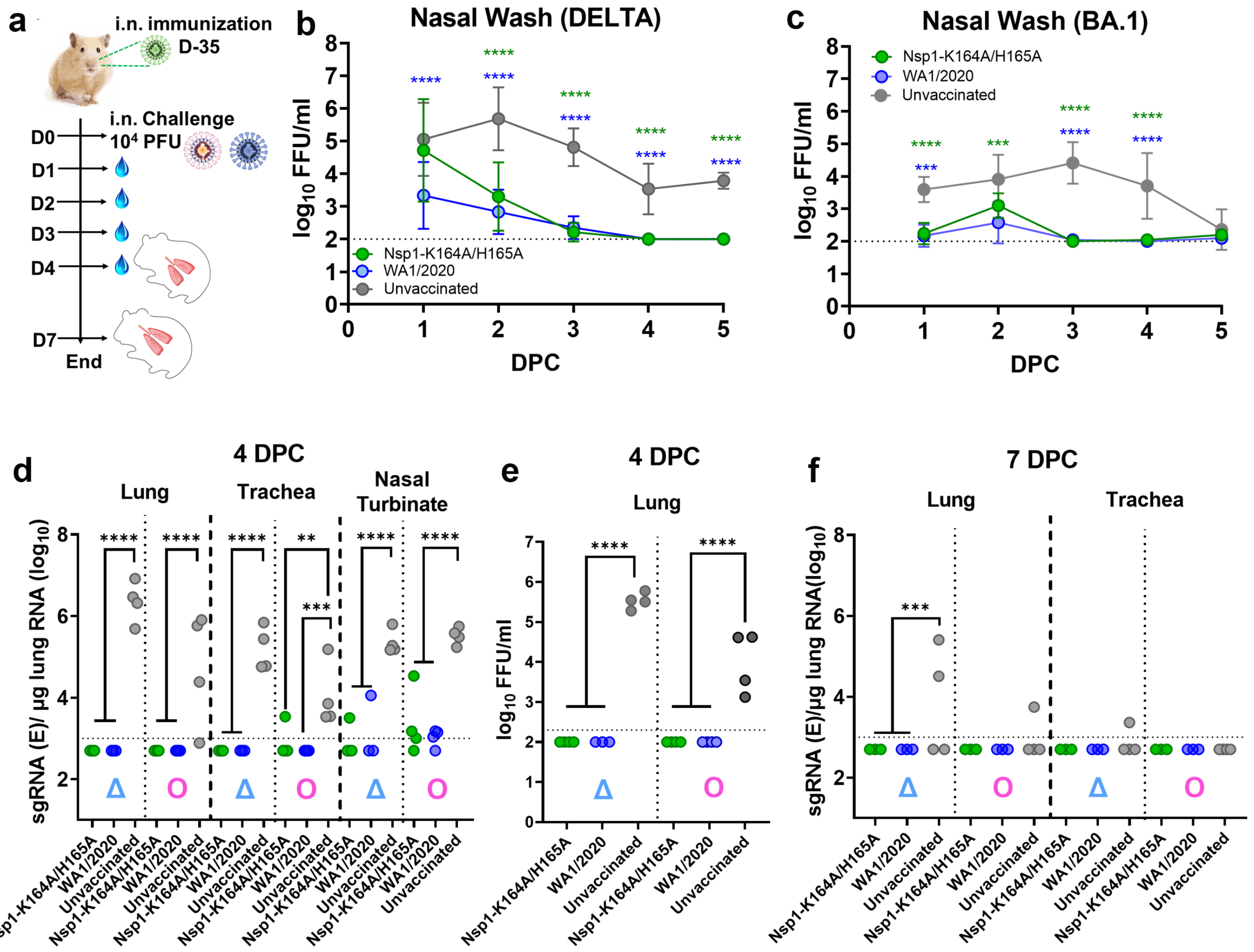

### Figure 3

**Intranasal immunization of Syrian hamsters with 100 PFU Nsp1-K164A/H165A significantly reduces viral loads in both upper and lower respiratory tract following challenge with Delta and Omicron variants. a** Syrian hamsters were vaccinated with 100 PFU Nsp1-K164A/H165A or infected with 100 PFU WA1/2020 35 days prior to challenge with $10^4$ PFU Delta (n=6) or BA.1 Omicron (n=7) isolates on day 0. **b-c** from 1-5 DPC, infectious virus from nasal wash samples was quantified by focus-forming assays in vaccinated, convalescent, or unvaccinated hamsters (n=8 for Delta, **b**; n=7 for Omicron BA.1, **c**). Graphs for (**b**) and (**c**) indicate mean values from a single experiment with standard deviations shown as error bars. **d** Viral sgRNA levels in lung, trachea, and nasal turbinate samples from 4 DPC (n=4 each, except WA1/2020-

Delta at n=3) were measured by qRT-PCR. **e** Infectious virus titers of lung homogenates at 4 DPC were determined by focus-forming assays. **f** Viral sgRNA levels in lungs and trachea at 7 DPC with Delta and BA.1 Omicron were measured by qRT-PCR. Dot plots represent samples collected from individual animals in a single experiment. Statistical differences were calculated using ordinary one-way analysis of variance (ANOVA) in GraphPad Prism 9.4.0 with Tukey's multiple comparisons tests. For statistical significance, ** indicates p<0.01, *** indicates p<0.001, and **** indicates p<0.0001. Δ: Delta variant challenge. O: Omicron BA.1 challenge. DPC: Days post-challenge.

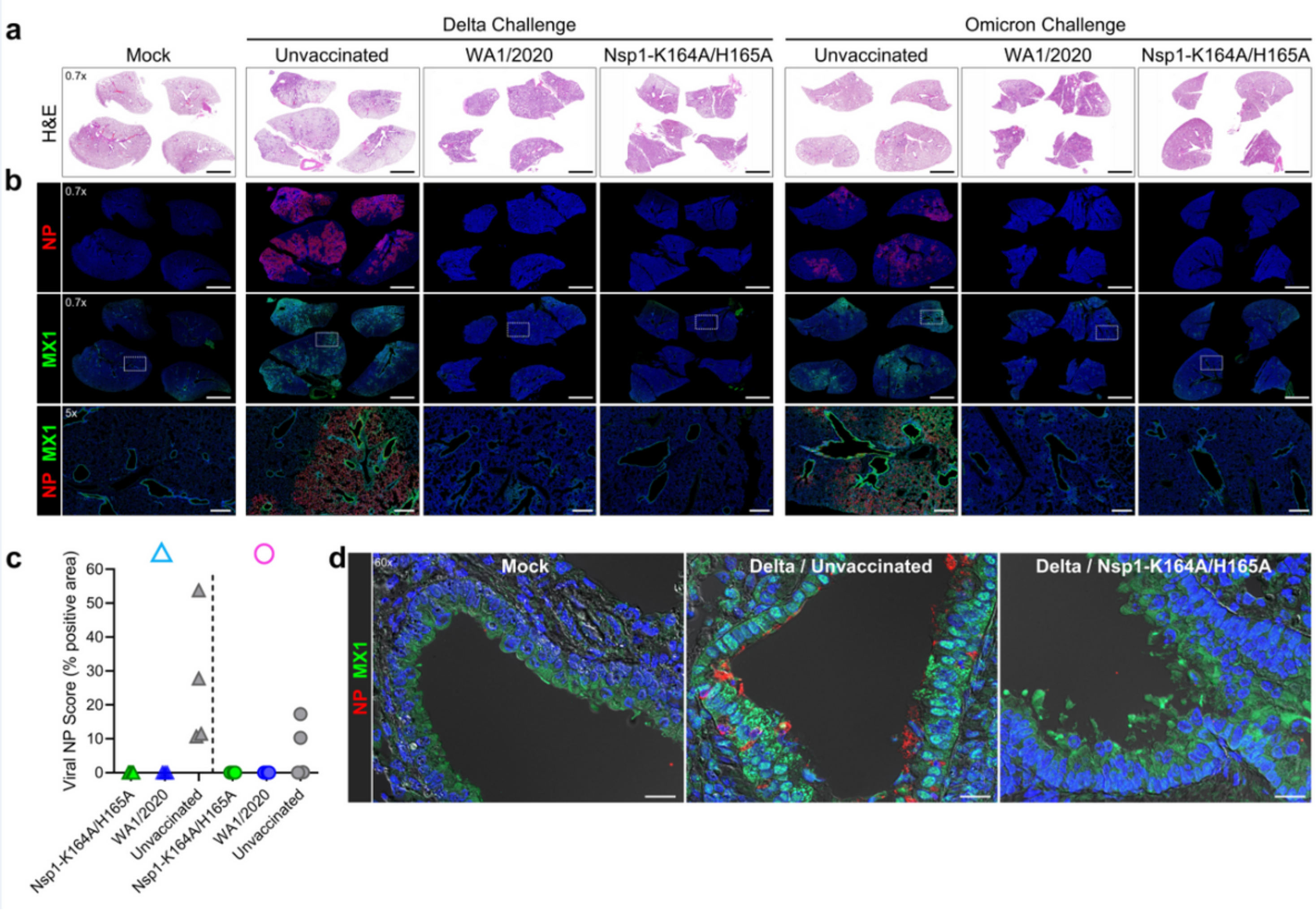

### Figure 4

#### Nsp1-K164A/H165A vaccination blocks virus propagation and MX1 induction in hamster lungs

Syrian hamsters were vaccinated with a low (100 PFU) dose of Nsp1-K164A/H165A or WA1/2020 35 days prior to challenge with a Delta or BA.1 isolate on day 0. Serial lung sections from non-infected non-vaccinated hamsters (mock) or hamster at 4 DPC were stained by (**a**) H&E or double-immunostained for (**b**) SARS-CoV-2 nucleocapsid protein (NP) and MX1 (interferon-induced antiviral protein). In (**a**), images are shown at one level of magnification (0.7x) while corresponding serial immunostained images in (**b**) are shown at two levels of magnification (0.7x and 5x) with white boxes delimiting the regions of magnification. **c** Semiquantitative analysis of viral NP staining in hamster lungs at 4 DPC. The plotted values represent the percent NP positive area as a function of the total lung area for each section (n = 3-4 animals per group). **d** High magnification immunofluorescence/differential interference contrast images of NP and MX1 in representative bronchioles of lung sections from mock hamsters or Delta-infected non-vaccinated or Nsp1-K164A/H165A-vaccinated hamsters at 4 DPC. Prominent cytoplasmic and nuclear localization of MX1 was detected in NP-positive bronchiolar epithelium in Delta-infected unvaccinated hamsters compared to low cytoplasmic expression of MX1 in mock and vaccinated hamsters. Nuclei were counterstained with Hoechst 33342 dye (blue). Scale bars: 5 mm (0.7x), 500 mm (5x), 20 mm (60x). Δ: Delta variant challenge. O: Omicron BA.1 challenge. DPC: Days post-challenge.

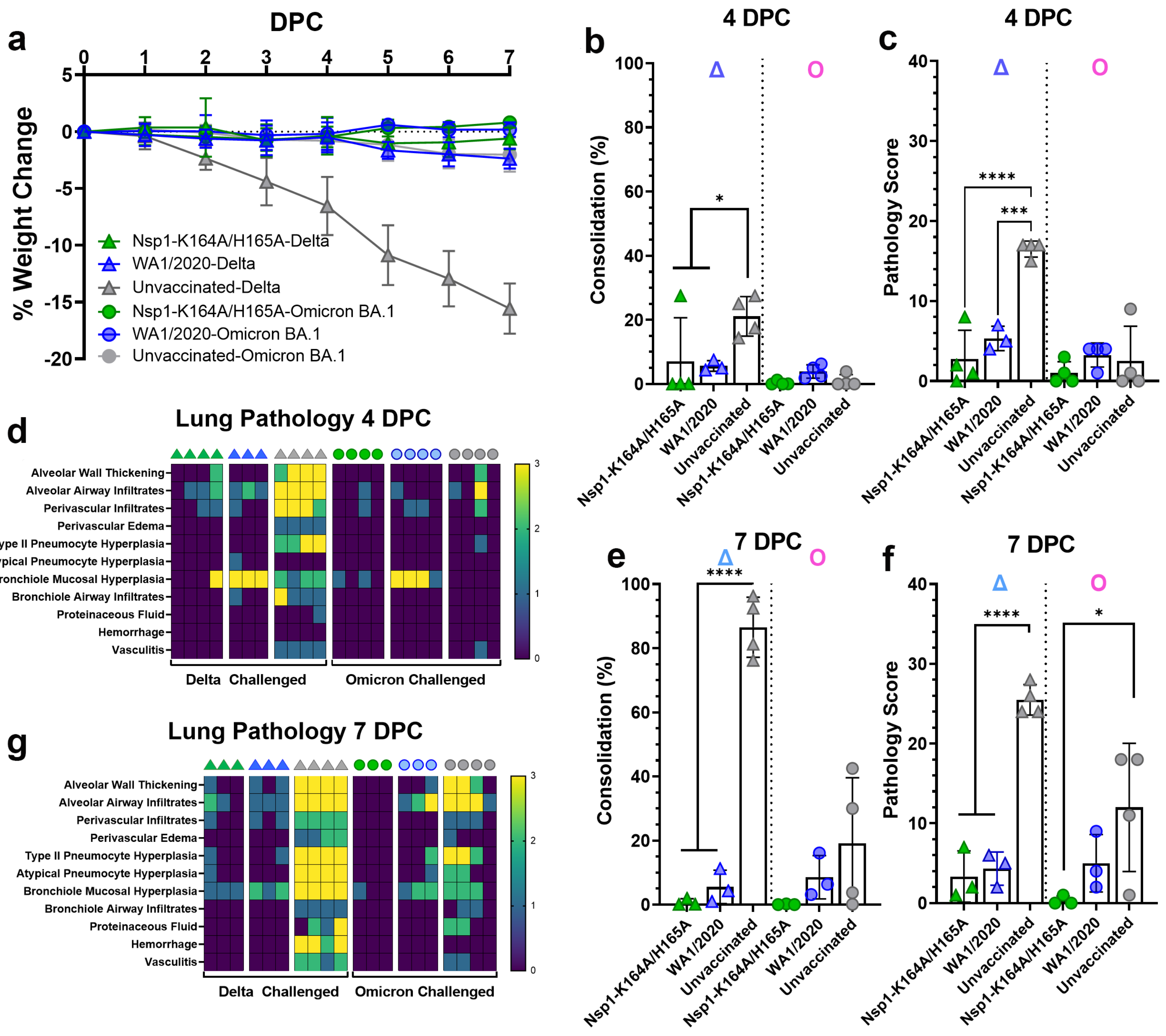

**Figure 5**

**Intranasal immunization of Syrian hamsters with Nsp1-K164A/H165A protects against Delta and Omicron challenge. a** Weight change was recorded for hamsters (described in Fig. 3a) after challenge by Delta and Omicron BA.1 variants for 7 days. Percentage of consolidation (**b**) and pathology score (**c**) in fixed lung tissues were compared between WA1/2020 convalescent (n=7), Nsp1-K164A/H165A vaccinated (n=8), and unvaccinated control (n=8) lungs at 4 DPC. Individual pathologies were graded by severity and presented in a heat map (**d**). Percentage of consolidation (**e**) and pathology score (**f**) were also compared at 7 DPC between WA1/2020 convalescent (n=6), Nsp1-K164A/H165A vaccinated (n=6), and unvaccinated control (n=8) lungs. **g** Heat-map presentation of individual pathologies at 7 DPC. Dot plots represent samples collected from individual animals in a single experiment. Statistical differences were calculated using ordinary one-way analysis of variance (ANOVA) in GraphPad Prism 9.4.0 with Tukey's multiple comparisons tests. For statistical significance, * indicates p<0.05, *** indicates p<0.001, and **** indicates p<0.0001. Δ:Delta variant challenge. O: Omicron BA.1 challenge. DPC: Days post-challenge.

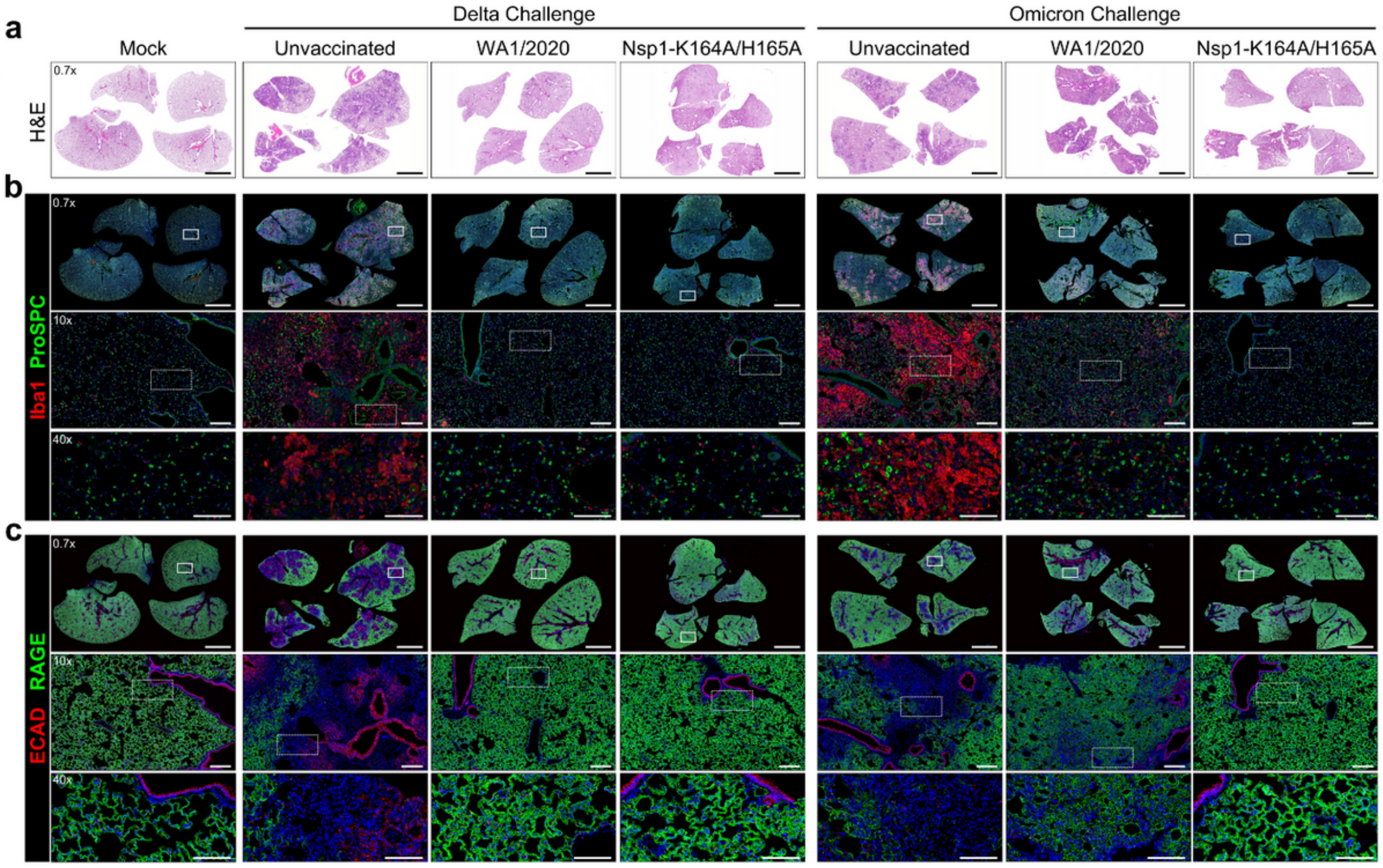

### Figure 6

**Nsp1-K164A/H165A vaccination protects against lung pathology post-challenge with Delta and Omicron isolates**

Syrian hamsters were vaccinated with a low (100 PFU) dose of Nsp1-K164A/H165A or WA1/2020 35 days prior to challenge with Delta or BA.1 Omicron isolates on day 0. Serial lung sections from non-infected non-vaccinated hamsters (mock) or 7 DPC-infected hamsters were stained by (**a**) H&E or double-immunostained for either (**b**) Iba1 (macrophage marker) and Prosurfactant protein C (ProSPC, AT2 marker) or (**c**) E-cadherin (ECAD, epithelial junctional marker) and RAGE (AT1 marker). Delta-infected unvaccinated lungs (n = 4) show extensive areas of tissue consolidation (**a**) that correspond with regions showing abundant Iba1-labeled macrophage accumulation and loss of ProSPC-labeled AT2 cells (**b**) as well as loss of alveolar wall RAGE-expressing AT1 epithelium surrounding affected ECAD-stained bronchioles and aberrant reepithelization (**c**). Similar though less extensive pathology was observed in two of four BA.1-challenged unvaccinated hamsters. Nsp1-K164A/H165A or WA1/2020 inoculation completely prevented or suppressed Delta or BA.1-induced lung pathology. In (**a**), images are shown at one level of magnification (0.7x) while corresponding serial immunostained images in (**b**) and (**c**) are shown at three levels of magnification (0.7x, 10x, and 40x) with white boxes delimiting the regions of magnification. Nuclei were counterstained with Hoechst 33342 dye (blue). Scale bars: 5 mm (0.7x), 250 mm (10x), 100 mm (40x).

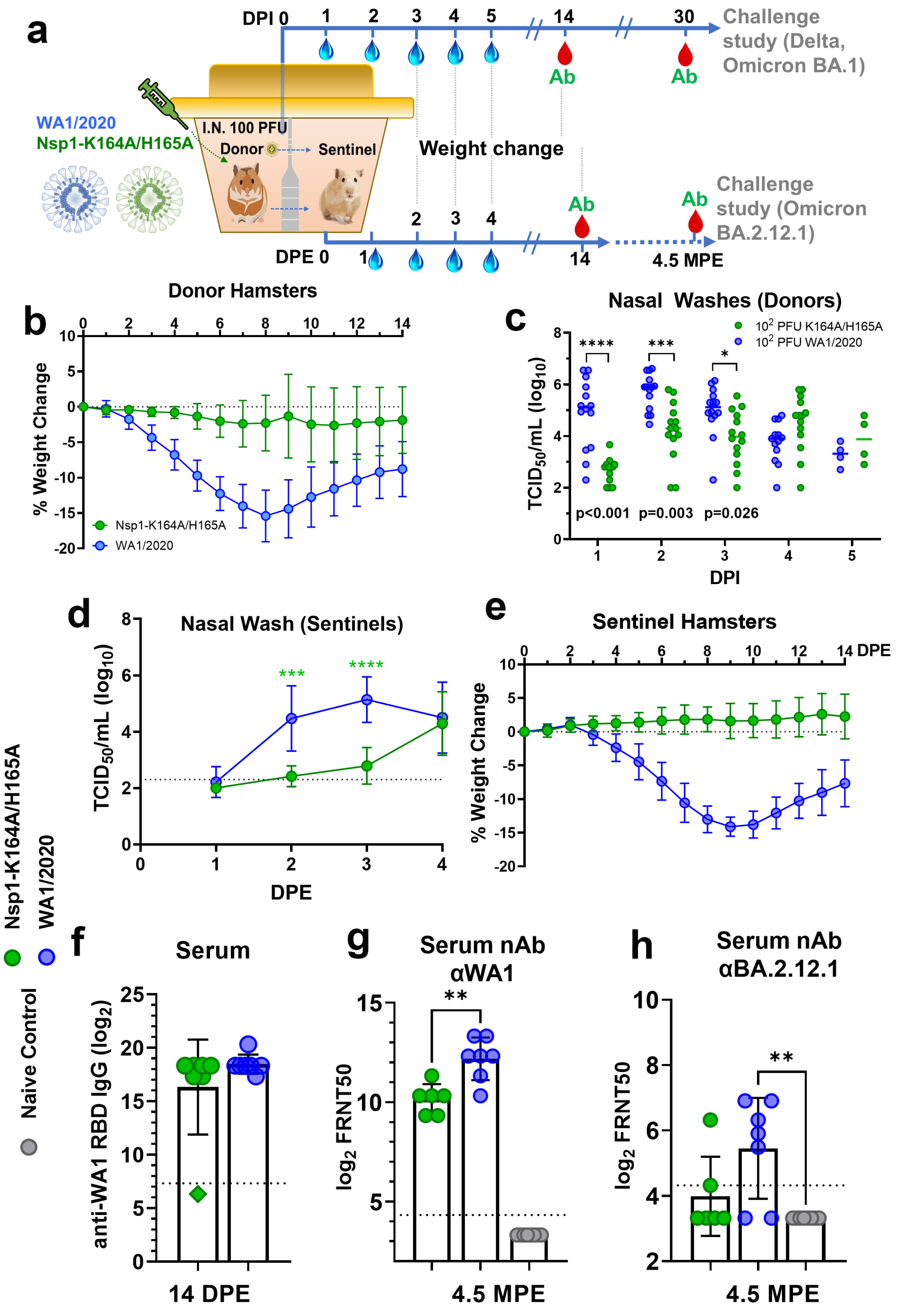

**Figure 7**

**Airborne transmission of Nsp1-K164A/H165A in Syrian hamsters**. **a** Donor Syrian hamsters (male, 5-month-old) were first inoculated with 100 PFU Nsp1-K164A/H165A (n=14) or WA1/2020 (n=14). One day after inoculation, donor hamsters were paired with recipient hamsters (sentinel, n=7/group) in the specially designed cages with metal dividers for monitoring airborne transmission. During pairing, nasal swabs were collected daily from sentinel hamsters for 4 days. **b** Weight loss profile of donor hamsters after virus inoculation. Symbols in (**b**) indicate mean percent weight change data of groups of hamsters (n=14) relative to initial individual animal weights on day 0. Statistical differences were calculated in GraphPad Prism 9.4.0 using a 2-way analysis of variance (ANOVA) with Sidak's multiple comparisons test. **c** Infectious virus titers of nasal wash samples collected from donor hamsters were measured by a $TCID_{50}$ assay for up to 5 days post-inoculation. Symbols indicate individual hamsters from a single experiment (n=14 per group). Statistical differences were calculated by Student's unpaired t-test in GraphPad Prism 9.4.0. **d** Infectious virus titers of nasal wash samples collected from sentinel hamsters were measured by a TCID50 assay for up to 4 days post-exposure (DPE) (n=7 per group). **e** Weight loss profile of sentinel hamsters (n=7 per group) up to 14 days post-exposure to donor hamsters. **f** Seroconversion of sentinel hamsters was confirmed by ELISA measuring anti-WA1/2020 RBD IgG. Note, one hamster (indicated by a green diamond) after exposure to Nsp1-K164A/H165A did not seroconvert. Serum nAB titers against WA1/2020 (**g**) and BA.2.12.1 (**h**) in sentinel hamsters (n=6 for sentinels exposed to Nsp1-K164A/H165A and N=7 for those exposed to WA/2020) after four and half months post-exposure (MPE) were measured by focus forming reduction neutralization assays.

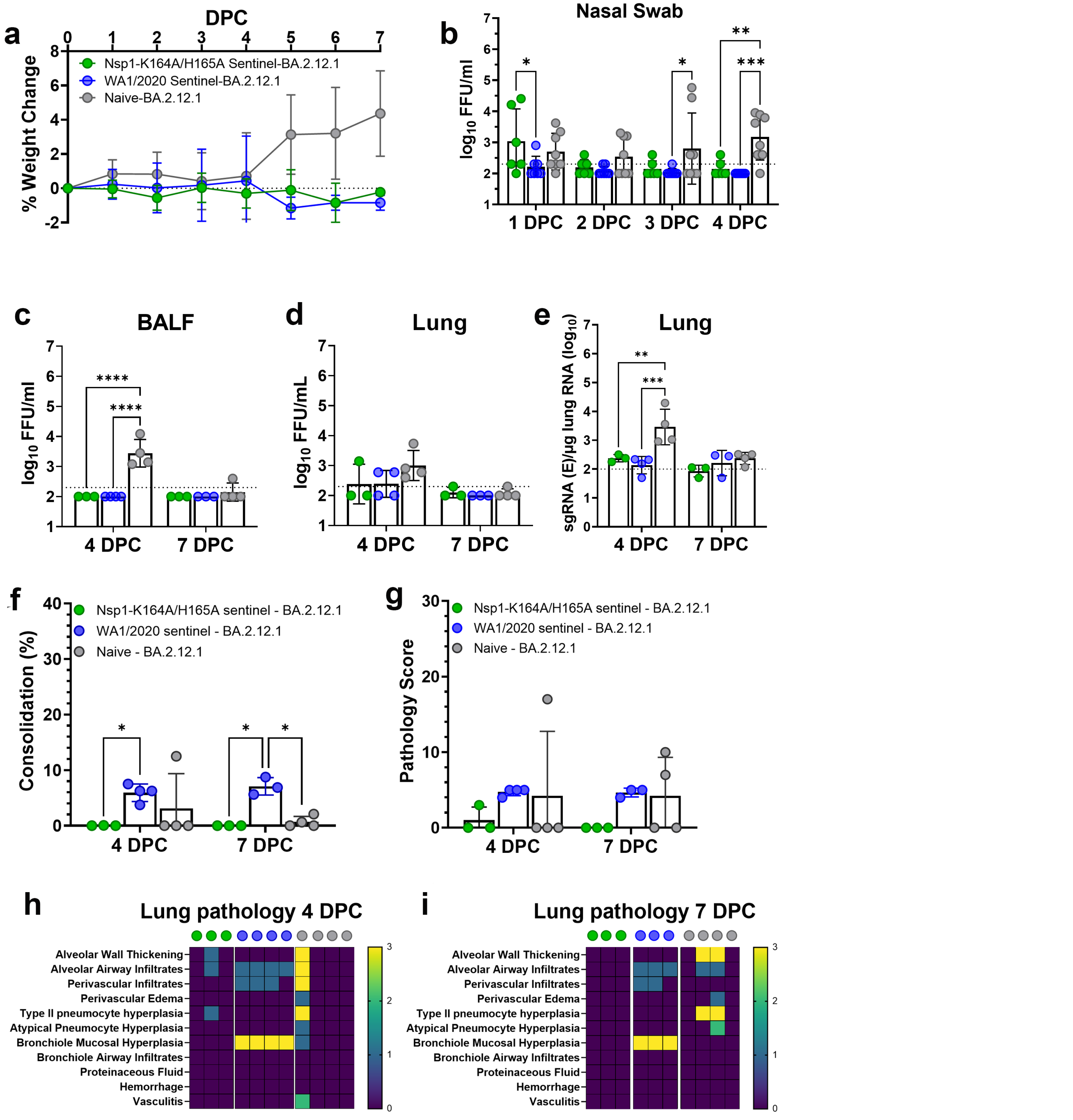

### Figure 8

**Seroconverted sentinel hamsters (i.e. passively vaccinated through transmission of Nsp1-K164A/H165A) are protected from BA.2.12.1 challenge. a** Seroconverted sentinel hamsters (4.5 months after exposure to WA1-2020 and Nsp1- K164A/H165A) were challenged with $10^4$ PFU of BA.2.12.1. A group of 8 age-matched naïve hamsters were also included in the challenge study as controls. Weight change was recorded for 7 days post-challenge. **b-d** Infectious virus titers from nasal swabs (**b**), BALF (**c**) and lung homogenates (**d**) were measured by focus-forming assays. **e** Viral sgRNA levels in the lungs were quantified by RT-pPCR 4 DPC and 7 DPC. Dot plots represent samples collected from individual animals in a single experiment. Percentage of consolidation (**f**) and pathology scores in lungs (**g**) of sentinel hamsters (n=6 for Nsp1-K164A/H165A, n=7 for WA1/2020, and n=8 for naïve controls) at 4 and 7 DPC with $10^4$ PFU of BA.2.12.1. Individual lung pathologies at 4 DPC (**h**) and 7 DPC (**i**) are presented in heat maps. Dot plots in this graph represent samples collected from individual animals in a single experiment. Statistical differences were calculated using ordinary one-way analysis of variance (ANOVA) in GraphPad Prism 9.4.0 with Tukey's multiple comparisons tests. For statistical significance, * indicates p<0.05, ** indicates p<0.01), *** indicates p<0.001, and **** indicates p<0.0001. DPC: Days post-challenge.

## Supplementary Files

This is a list of supplementary files associated with this preprint. Click to download.

- MovieS1.mp4
- SupplementaryMaterials.pdf
